# Supplementary material for: Risk Assessment of Passive Smoking Based on Analysis of Hair Nicotine and Cotinine as Exposure Biomarkers by In-Tube Solid-Phase Microextraction Coupled On-Line to LC-MS/MS
Source: Molecules. 2021 Dec 3;26(23):7356. doi: 10.3390/molecules26237356 (PMC8659248; doi:10.3390/molecules26237356)
Supplement: Supplementary file 1 [file molecules-26-07356-s001.zip › molecules-1494352-supplementary.pdf]

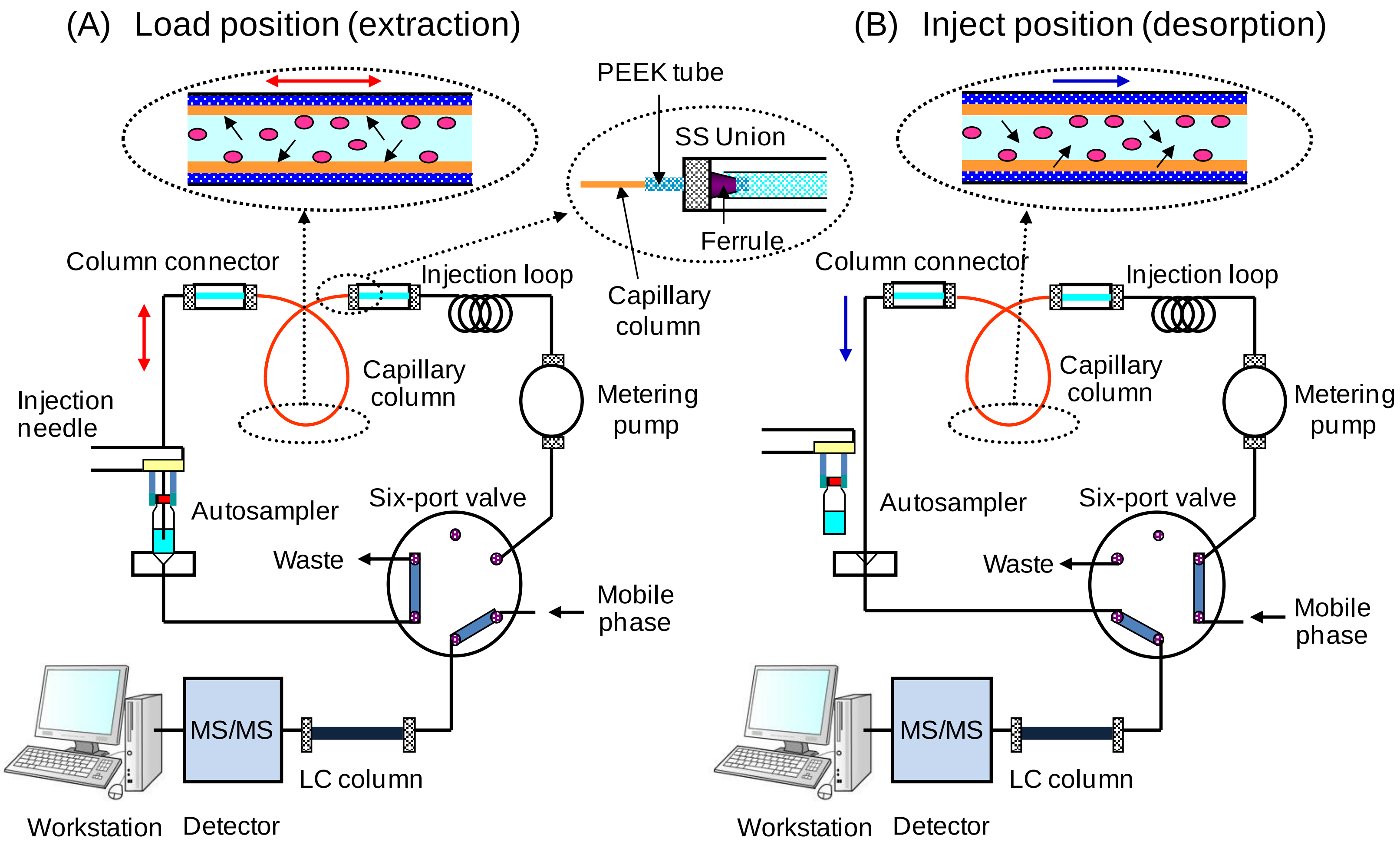

Figure S1. Outline of online in-tube SPME LC-MS/MS system (**A**) load position (extraction) and (**B**) inject position (desorption).

Table S1. Linearity, sensitivity and precisions of nicotine and cotinine by in-tube SPME LC–MS/MS.

Compound	Linearity		LOD [2] (pg mL^{-1})		Concentration (pg mL^{-1})	Precision (RSD [3] %), (n = 5)	
	Range (pg mL^{-1})	Correlation coefficient [1]	Direct injection	In-tube SPME		Intra-day	Inter-day
**Nicotine**	5–1000	0.9997	8.56	0.45	20	1.62	2.42
					200	1.64	5.97
**Cotinine**	5–1000	0.9992	2.68	0.13	20	3.37	2.35
					200	3.12	2.49

[1] n = 24. [2] Limits of detection (signal to noise ratio of 3). [3] RSD, relative standard deviation.

Table S2. Program for the in-tube SPME process.

Sequence	Event	Switching valve	Vial	Draw / Ejection		
				Cycle [1]	Volume (μL)	Speed (μL min^{-1})
1	Conditioning of the capillary	Load	MeOH	D/E (2)	40	200
2	Drawing of air into the capillary	Load	Empty	D (1)	50	200
3	Conditioning of the capillary	Load	Water	D/E (2)	40	200
4	Extraction of analytes into the capillary	Load	Sample	D/E (20)	40	200
5	Needle washing	Load	MeOH	D/E (1)	2	200
6	Desorption of analytes from the capillary	Inject	–	–	–	–
7	HPLC separation of analytes and return to sequence 1	Load	–	–	–	–

[1] D: draw, E: ejection.

Table S3. MS/MS transitions and setting parameters for nicotine, cotinine and their stable isotope-labeled compounds.

Compound	Mass transition (m/z)	Dwell time (msec)	DP (V)	EP (V)	CE (eV)	CXP (V)
Nicotine (Quantifier)	163.1 → 132.1	166.5	60	5	20	10
Nicotine (Qualifier)	163.1 → 106.1	166.5	60	10	30	10
Nicotine-d_3	166.1 → 132.0	166.5	60	5	20	10
Cotinine (Quantifier)	177.1 → 80.2	166.5	70	15	40	15
Cotinine (Qualifier)	177.1 → 98.1	166.5	60	10	30	10
Cotinine-d_3	180.1 → 80.1	166.5	70	15	40	15
